# UBE2M Identified by CRISPR Screening as a Key Regulator of Cisplatin-Induced Acute Kidney Injury *via* the p53 Pathway

**DOI:** 10.2174/0118715303410982251112055248

**Published:** 2026-03-12

**Authors:** Cheng Yuan, Feng Chen, Xueyun Gao, Ayinigaer Yusufu, Danqin Lu, Xiaoyan Wu, Lihua Ni

**Affiliations:** 1 Department of Oncology, Yichang Central People's Hospital and The First College of Clinical Medical Science, China Three Gorges University, Yichang, Hubei, China;; 2 Tumor Prevention and Treatment Center of Three Gorges University and Cancer Research Institute of Three Gorges University, Yichang, Hubei, China;; 3 Clinical Medical Research Center for Precision Diagnosis and Treatment of Lung Cancer and Management of Advanced Cancer Pain of Hubei Province, Wuhan, China;; 4 Department of Nephrology, Zhongnan Hospital of Wuhan University, Wuhan, Hubei, 430071, China;; 5 Department of Critical Care Medicine, Jingzhou Central Hospital, Jingzhou, Hubei, 434000, China;; 6 Department of General Practice, Zhongnan Hospital of Wuhan University, Wuhan, Hubei, 430071, China

**Keywords:** Cisplatin, UBE2M, acute kidney injury (AKI), p53, apoptosis, renal tubular epithelial cells

## Abstract

**Introduction:**

Acute kidney injury caused by cisplatin (Cis-AKI) is a major limitation in its clinical use, primarily due to the lack of effective therapeutic targets to mitigate nephrotoxicity. Although several molecular pathways are involved in Cis-AKI, identifying reliable and actionable therapeutic targets has been challenging. Through a CRISPR-based genome-wide screening approach, UBE2M was identified as a novel gene involved in cellular survival during cisplatin-induced stress. However, its expression, biological function, and underlying mechanism in Cis-AKI have not been thoroughly investigated. This study aims to identify key therapeutic targets for Cis-AKI and investigate the role of UBE2M in this condition.

**Methods:**

A CRISPR-Cas9 genome-wide screening approach was employed to identify key genes involved in cisplatin-induced renal tubular epithelial cell injury. UBE2M, identified as a critical survival factor, was further investigated using both gain- and loss-of-function strategies to explore its biological function and underlying regulatory mechanisms in the Cis-AKI model

**Results:**

CRISPR screening identified UBE2M as a key regulator of cellular survival in Cis-AKI, and subsequent validation experiments confirmed its suppression in cisplatin-induced renal injury models. UBE2M overexpression alleviated apoptosis and renal injury by reducing p53 activation. In contrast, UBE2M knockdown exacerbated these effects, leading to increased apoptosis and renal injury.

**Discussion:**

This study reveals that UBE2M is a critical regulator of cisplatin-induced renal tubular epithelial cell injury. By regulating the p53-mediated apoptotic pathway, UBE2M protects against Cis-AKI.

**Conclusion:**

UBE2M could serve as a novel therapeutic target for the prevention and treatment of cisplatin-induced nephrotoxicity.

## 1 INTRODUCTION

Cisplatin, as a chemotherapeutic agent widely used in the treatment of various solid tumors, is severely affected by dose-limiting nephrotoxicity in clinical applications. Acute kidney injury induced by cisplatin (Cis-AKI) is one of its major side effects, with about 30% of patients developing AKI after receiving cisplatin [1, 2]. Even those who do not progress to AKI still face the risk of long-term renal function decline and the development of chronic kidney disease (CKD) [3, 4]. Therefore, it is crucial to elucidate molecular mechanisms that bridge these pathogenic processes and to identify novel therapeutic targets for precision intervention.

In this study, we employed an unbiased, genome-wide CRISPR-Cas9 screening approach targeting E1, E2, and E3 ubiquitination enzymes in HK-2 cells to identify key regulators involved in cisplatin-induced renal tubular injury. This high-throughput screening led to the discovery of UBE2M, an E2 ligase, as a previously unrecognized critical mediator of tubular damage caused by cisplatin. Previous research has suggested that UBE2M participates in regulating various cellular pathways, including those governing cell proliferation and survival. For instance, studies in cancer models have indicated that UBE2M modulates tumor cell proliferation and resistance to apoptosis through its regulation of the cell cycle [5]. Moreover, UBE2M has been shown to interact with key signaling pathways, such as HIF-1α [6] and Nrf2 [7], which are crucial for cellular responses to oxidative stress and inflammation, both of which are involved in cisplatin-induced kidney injury [8-10]. Despite these findings, the role of UBE2M in renal tubular epithelial cell injury, especially under cisplatin-induced stress, remains unexplored.

Given the established involvement of p53, a master regulator of cellular stress responses and apoptosis [11-13], we sought to investigate whether UBE2M exerts its protective effects through modulation of the p53 pathway. Emerging evidence suggests that modulation of p53 activity can significantly influence the severity and outcomes of Cis-AKI [14-17]. This relationship is particularly critical to understand, as p53-mediated apoptosis represents a central convergence point of oxidative stress, inflammation, and metabolic dysregulation in kidney injury. Targeting this molecular axis may offer new precision therapeutic strategies tailored to the complex pathophysiology of Cis-AKI.

In summary, through an integrated CRISPR-based functional screening and mechanistic validation approach, we identified UBE2M as a novel regulator of Cis-AKI, functioning in part *via* suppression of p53-mediated apoptotic signaling. Our findings not only provide new insights into the molecular crosstalk underpinning cisplatin-induced renal injury but also highlight UBE2M as a promising therapeutic target for precision intervention in chemotherapy-associated kidney damage.

## 2 MATERIALS AND METHODS

### 2.1 Study Design

This study combined *in vitro* and *in vivo* experimental approaches. For the *in vitro* component, a genome-wide CRISPR-Cas9 screening was conducted in HK-2 cells to identify key genes involved in cisplatin-induced cytotoxicity. The screening library targeted E1, E2, and E3 ubiquitination-related genes. Surviving cells post cisplatin treatment were analyzed by next-generation sequencing to identify protective or sensitizing gene candidates, among which UBE2M was selected for further investigation.

For the *in vivo* component, an AKI model was established in male C57BL/6J mice (8-10 weeks old) *via* a single intraperitoneal injection of cisplatin (20 mg/kg). Twelve mice were randomly assigned to control and treatment groups. Outcome measurements included renal histology, TUNEL staining, ROS detection, immunohistochemistry, and Western blotting.

Investigators were aware of group allocation during assignment and experiment conduct, but outcome assessment and data analysis were performed blinded to group allocation. Statistical analysis was performed using GraphPad Prism. All experiments were performed independently, at least in triplicate. Data were expressed as mean ± SD.

### 2.2 CRISPR Screening

To identify key genes involved in Cis-AKI, a CRISPR-Cas9-based genetic screen was performed using a library comprising approximately 3,380 sgRNAs targeting around 845 genes. The sgRNA library was delivered into human renal proximal tubular epithelial cells (HK-2) *via* lentiviral transduction. Cells were infected at a multiplicity of infection (MOI) of 0.3 to ensure that each cell incorporated a single guide RNA (sgRNA). Puromycin (2 µg/mL) was applied for 72 hours to eliminate non-transduced cells. After expansion to achieve 1,000× coverage of the library, genomic DNA was extracted, and deep sequencing was performed to evaluate sgRNA integration efficiency.

Following successful library construction, HK-2 cells were treated with cisplatin (20 µM) for 24 hours to induce acute kidney injury. Genomic DNA was extracted from surviving cells and subjected to next-generation sequencing (NGS) to identify sgRNAs enriched during cisplatin-induced stress. Raw sequencing data were processed to calculate the relative abundance of each sgRNA before and after selection. Enriched sgRNAs in the surviving population were identified by comparing sequencing data to the reference library.

Data analysis was performed using MAGeCK (Model-based Analysis of Genome-wide CRISPR-Cas9 Knockout) and DESeq2 to identify candidate genes whose knockout conferred resistance or sensitivity to cisplatin-induced AKI. Genes with significant changes in abundance (log_2_ fold change > 1 or < -1) and high enrichment scores were considered candidate genes.

### 2.3 Cell Culture and Treatments

HK-2 were cultured in Minimum Essential Medium (MEM, Pricella, China, PM150410) with 10% FBS. Cells were treated with cisplatin (20 µM) for 24 hours to induce injury. Negative controls were treated with DMSO. UBE2M expression was manipulated using siRNA (si-UBE2M) or overexpression plasmids (OE-UBE2M). Transfections were performed using Lipofectamine 3000 according to the manufacturer’s protocol. Details of the siRNA and overexpression virus used for UBE2M upregulation and downregulation *in vitro* are provided in Table **1**. The p53 inhibitor (Pifithrin-α hydrobromide, MCE, 20 μM) and p53 activator (NSC-207895, MCE, 0.5 μM) were co-cultured to investigate whether the p53 pathway mediates the functional role of UBE2M in cisplatin-induced injury.

### 2.4 RNA Extraction and qPCR

Total RNA was extracted from cells using TRIzol reagent and reverse transcribed into cDNA using a reverse transcription kit. Gene expression levels of UBE2M, KIM-1, NGAL, and GAPDH were quantified by qPCR using SYBR Green Master Mix. Primer sequences are listed in Table **2**.

### 2.5 Western Blot Analysis

Protein samples were extracted using RIPA lysis buffer containing protease inhibitors. Equal amounts of protein were separated by SDS-PAGE, transferred to PVDF membranes, and probed with antibodies against UBE2M, p53, KIM-1, NGAL, Bax, Bcl-2, GPX4, ACSL4, and GAPDH. Signals were detected using enhanced chemiluminescence (ECL) reagents, and band intensities were quantified using ImageJ. Details of all antibodies and experimental conditions are provided in Table **3**.

### 2.6 TUNEL Assay

Apoptotic cells were detected using a TUNEL apoptosis detection kit. After treatment, cells were fixed, permeabilized, and labeled with the TUNEL reaction mixture. Nuclei were counterstained with DAPI, and apoptotic cells (TUNEL-positive) were visualized using fluorescence microscopy. Quantification was performed by calculating the percentage of TUNEL-positive nuclei relative to total nuclei.

### 2.7 Immunofluorescence Staining

Cells were fixed with 4% paraformaldehyde, permeabilized, and incubated with primary antibodies against UBE2M and KIM-1, followed by fluorescently labeled secondary antibodies. Nuclei were stained with DAPI. Fluorescence images were captured using a light microscope, and the relative fluorescence intensity was quantified using ImageJ.

### 2.8 ROS Detection

Intracellular ROS levels were measured using DHE (dihydroethidium) staining. Cells were incubated with DHE dye for 30 minutes at 37°C in the dark and imaged using fluorescence microscopy. ROS levels were quantified by measuring the fluorescence intensity.

### 2.9 Animal Studies

Male C57BL/6 mice, aged 8 to 10 weeks and weighing approximately 20 g, were purchased from Wuhan Wanqian Jiahe Experimental Animal Breeding Center. A Cis-AKI mouse model was established by intraperitoneal injection of cisplatin (20 mg/kg) in a total of 6 male C57BL/6J mice. Control mice (n = 6) received an equal volume of saline by intraperitoneal injection. Mice were randomly allocated to control and cisplatin treatment groups using a simple random number method. All animals were housed under standard conditions with controlled temperature, humidity, and a 12-hour light/dark cycle, with free access to food and water. Potential confounders were minimized by housing animals under identical environmental conditions and by performing treatments and outcome assessments in a consistent order across groups. Cage locations were rotated regularly to avoid position effects. Health monitoring was performed regularly, and humane endpoints were established to minimize suffering. Mice were sacrificed after 72 hours, and kidney tissues were collected for histological and molecular analyses.

### 2.10 Histological Analysis

Kidney tissues were fixed, embedded, and sectioned for histological evaluation. HE staining was used to assess tubular necrosis and injury. Masson’s trichrome staining quantified collagen deposition and fibrosis, while PAS staining evaluated glycogen accumulation and basement membrane integrity. Histological changes were imaged under a light microscope and quantified by blinded investigators. Immunohistochemistry (IHC) was performed to detect UBE2M expression in kidney tissues. TUNEL staining was used to identify apoptotic cells *in situ.*

### 2.11 Cell Viability Assay

Cell viability was assessed using the Cell Counting Kit-8 (CCK-8, Abbkine, America, BMU106-CN) according to the manufacturer's instructions. Briefly, HK2 cells were seeded into 96-well plates at a density of 5 × 10^3^ cells/well and treated with cisplatin or other reagents for 24 hours. After treatment, 10 μL of CCK-8 solution was added to each well, and the plates were incubated at 37°C for 2 hours. The absorbance at 450 nm was measured using a microplate reader. Cell viability was calculated as a percentage relative to the control group.

### 2.12 Statistical Analysis

All experiments were performed at least in triplicate, independently. Data are presented as mean ± SD. Quantitative data following a normal distribution were presented as mean ± standard deviation (Mean ± SD). One-way and two-way analysis of variance (ANOVA) were used for comparisons among multiple groups, while the t-test was applied for comparisons between two groups. A *P*-value less than 0.05 was considered statistically significant.

## 3 RESULTS

### 3.1 CRISPR Screening Reveals the Key Role of UBE2M in Cisplatin-Induced Renal Tubular Cell Injury

**Fig. (1):**
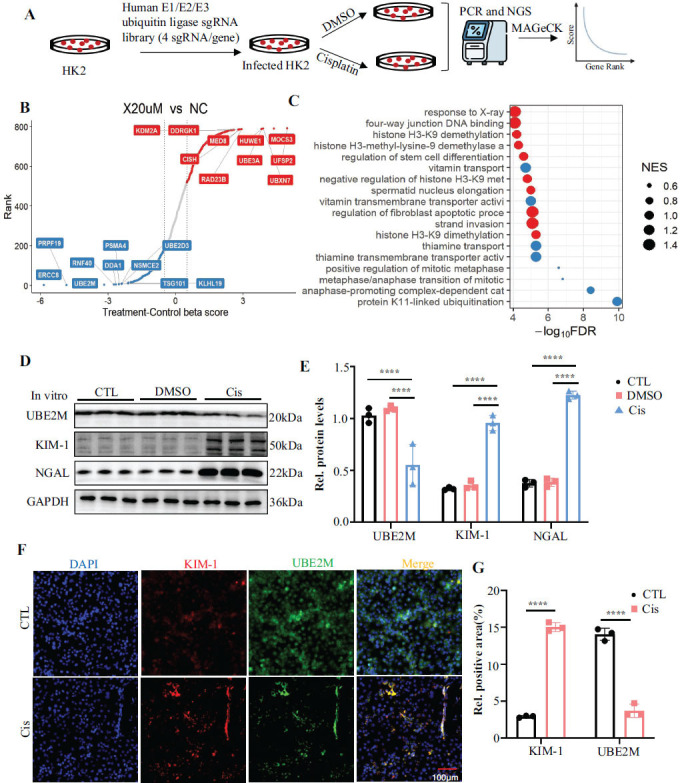
CRISPR screening reveals the key role of UBE2M in cisplatin-induced renal tubular cell injury. (**A**) Diagram illustrating the CRISPR-Cas9 screening process. HK-2 cells were infected with sgRNAs targeting a human E1/E2/E3 ubiquitin ligase gene library, followed by 24-hour treatment with either DMSO or cisplatin (Cisplatin). PCR amplification and NGS sequencing were performed, and key genes were identified through MAGeCK software analysis. (**B**) Beta score analysis showing the distribution of candidate genes affected by cisplatin treatment. UBE2M was identified as a significantly downregulated gene. (**C**) Gene Set Enrichment Analysis (GSEA) of pathways enriched among the differentially expressed genes following cisplatin treatment. (**D**) Western blot analysis of UBE2M, KIM-1, and NGAL expression in HK-2 cells treated with DMSO or cisplatin. GAPDH served as a loading control. (**E**) Quantification of protein expression levels relative to GAPDH (n = 3 independent experiments). (**F**) Representative immunofluorescence images showing UBE2M (green) and KIM-1 (red) expression in HK-2 cells treated with or without cisplatin. Nuclei were counterstained with DAPI (blue). Scale bar: 100 μm. (**G**) Quantification of the positive staining area for UBE2M and KIM-1 (n = 3). Data are presented as mean ± SD. ****P < 0.0001.

To identify genes integral to cisplatin-induced injury in renal tubular epithelial cells, this study employed CRISPR-Cas9 technology to develop an HK-2 cell library specifically targeting genes encoding ubiquitin-activating enzyme E1, ubiquitin-conjugating enzyme E2, and ubiquitin ligase E3. The HK-2 cells were exposed to either DMSO or cisplatin for 24 hours to induce cellular injury. After treatment, surviving cells were collected for next-generation sequencing (NGS) analysis, and the sgRNA abundance was evaluated using the MAGeCK algorithm (Fig. **1A**).

The beta score analysis revealed multiple candidate genes whose loss conferred differential survival upon cisplatin exposure. Among them, UBE2M exhibited a significant decrease in beta score under cisplatin treatment compared to control, suggesting that UBE2M knockdown may affect cell susceptibility to cisplatin-induced injury (Fig. **1B**). Gene expression profiling (Fig. **1C**) was followed by Gene Set Enrichment Analysis (GSEA), showing enrichment of pathways associated with DNA damage response, histone modification, and ubiquitination processes subsequent to cisplatin exposure. Furthermore, western blotting (Figs. **1D** and **E**) confirmed that UBE2M expression was significantly decreased in cisplatin-treated cells, while the markers of renal injury, KIM-1 and NGAL, were increased. Immunofluorescence staining (Figs. **1F** and **G**) demonstrated a marked decrease in UBE2M-positive areas and an increase in KIM-1-positive areas in cisplatin-treated cells, supporting their role in the progression of renal injury. These findings suggest that UBE2M plays an important role in the pathophysiology of cisplatin-induced kidney injury and may be a potential therapeutic target for mitigating renal damage.

### 3.2 Downregulation of UBE2M Expression in a Mouse Model of Cisplatin-Induced Kidney Injury

**Fig. (2):**
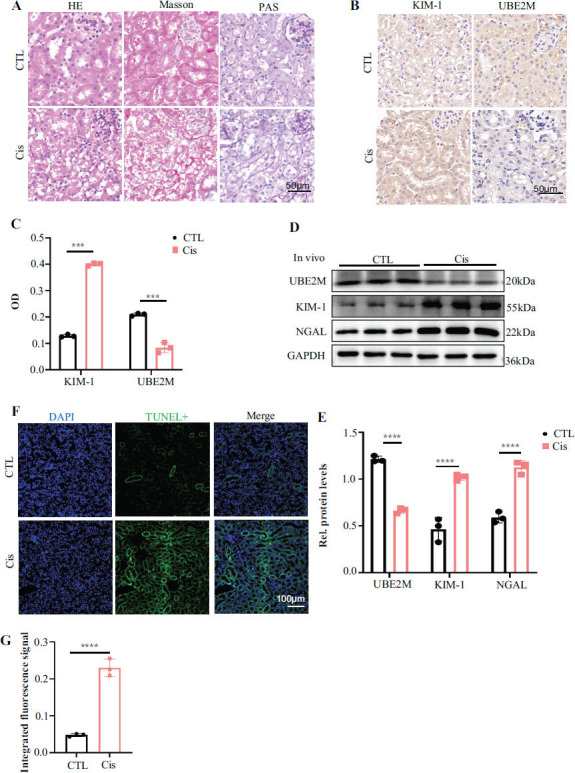
Cisplatin-induced acute kidney injury (AKI) is associated with decreased UBE2M expression and increased tubular injury. (**A**) Representative images of kidney histology from control (CTL) and cisplatin (Cis)-treated mice. Hematoxylin-eosin (HE), Masson’s trichrome, and periodic acid-Schiff (PAS) staining were performed to evaluate tubular injury and fibrosis. Scale bar: 50 μm. (**B**) Immunohistochemical analysis of KIM-1 and UBE2M protein expression in kidney tissue. (Scale bar: 50 μm). (**C**) Quantification of optical density (OD) values for KIM-1 and UBE2M immunostaining (n = 3). (**D**) Western blot analysis of UBE2M, KIM-1, and NGAL protein levels in renal tissues from control and cisplatin-treated mice. GAPDH served as the loading control. (**E**) Quantification of relative protein levels normalized to GAPDH (n = 3). (**F**) TUNEL staining of kidney sections showing apoptotic cells (green) and nuclei counterstained with DAPI (blue). Scale bar: 100 μm. (**G**) Quantification of integrated TUNEL fluorescence signal (n = 3). Data are expressed as mean ± SD. ***P < 0.001, ****P < 0.0001.

To examine the expression changes of UBE2M in a cisplatin-induced kidney injury model, a cisplatin treatment mouse model was established, followed by histopathological and molecular level analyses. As shown in Fig. (**2A**), Hematoxylin and Eosin (HE) staining revealed significant damage to renal tubular epithelial cells in the Cis group, characterized by cell swelling, necrosis, and tubular dilation. Masson staining demonstrated significant fibrosis in the renal interstitium, while PAS staining further showed disrupted renal tubular basement membrane structure in the cisplatin-treated group, which was notably more severe compared to the control group (CTL). Immunohistochemical analysis, as shown in Figs. (**2B** and **C**), revealed a marked increase in KIM-1 expression and a significant decrease in UBE2M expression in the Cis group. Western blot results Figs. (**2D** and **E**) further confirmed that KIM-1 and NGAL protein levels were significantly elevated in the Cis group, whereas UBE2M protein expression was clearly decreased compared to the CTL group. Furthermore, TUNEL staining (Figs. **2F**-**G**) demonstrated that cisplatin treatment significantly increased the number of apoptotic tubular cells, as indicated by stronger TUNEL-positive fluorescence in the Cis group relative to the CTL group, suggesting enhanced tubular cell apoptosis during cisplatin-induced kidney injury.

In summary, cisplatin-induced kidney tissue injury is characterized by structural damage to renal tubular epithelial cells, increased interstitial fibrosis, and a significant downregulation of UBE2M protein, suggesting that UBE2M may play an important role in the process of cisplatin-induced kidney injury.

### 3.3 Overexpression of UBE2M Alleviates Cisplatin-Induced Injury in Renal Tubular Epithelial Cells

**Fig. (3):**
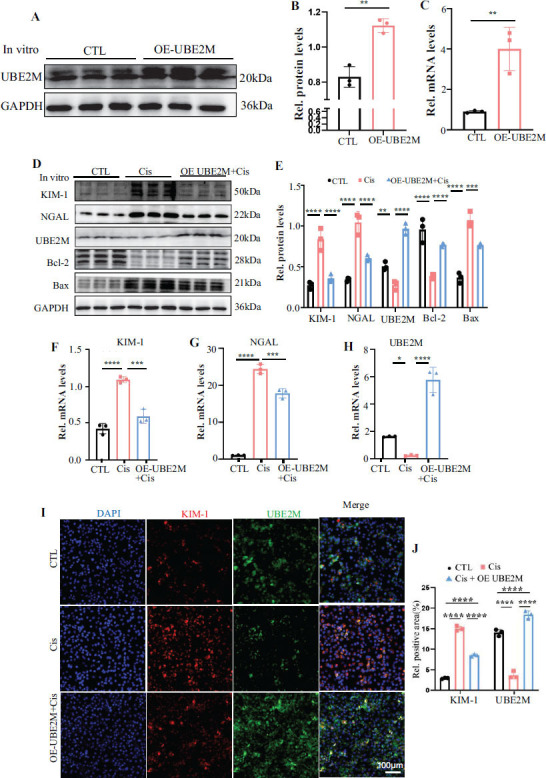
Overexpression of UBE2M alleviates cisplatin-induced injury in HK-2 cells. (**A**) Western blot analysis showing UBE2M protein expression in control (CTL) and UBE2M-overexpressing (OE-UBE2M) HK-2 cells. GAPDH served as a loading control. (**B-C**) Quantification of UBE2M protein (**B**) and mRNA (**C**) levels in CTL and OE-UBE2M groups (n = 3). (**D**) Western blot analysis of KIM-1, NGAL, UBE2M, Bcl-2, and Bax in HK-2 cells treated with CTL, cisplatin (Cis), or OE-UBE2M plus cisplatin (OE-UBE2M+Cis). (**E**) Quantification of protein levels normalized to GAPDH (n = 3). (F-H) Quantification of mRNA levels of KIM-1 (**F**), NGAL (**G**), and UBE2M (**H**) under the indicated conditions (n = 3). (**I**) Representative immunofluorescence images of HK-2 cells stained for KIM-1 (red) and UBE2M (green) with DAPI counterstaining (blue) in CTL, Cis, and OE-UBE2M+Cis groups. Scale bar: 200 μm. (**J**) Quantification of the positive staining areas for UBE2M and KIM-1 (n = 3). Data are presented as mean ± SD. *P < 0.05, **P < 0.01, ***P < 0.001, ****P < 0.0001.

To investigate the role of UBE2M in renal tubular cell injury, both gain- and loss-of-function approaches were employed. First, UBE2M overexpression was validated at the protein and mRNA levels (Figs. **3A**-**C**), confirming successful transfection. Cisplatin-induced renal injury markers, including KIM-1 and NGAL, were markedly upregulated, while anti-apoptotic Bcl-2 was suppressed and pro-apoptotic Bax was elevated. However, UBE2M overexpression significantly attenuated these changes, as shown by Western blot and qPCR analyses (Figs. **3D**-**H**). Immunofluorescence further revealed reduced KIM-1 expression and enhanced UBE2M levels in the OE-UBE2M + Cis group (Fig. **3I**-**J**), supporting a protective role of UBE2M in cisplatin-induced injury.

### 3.4 Silencing UBE2M Aggravates Cisplatin-Induced Injury in Renal Tubular Epithelial Cells

**Fig. (4):**
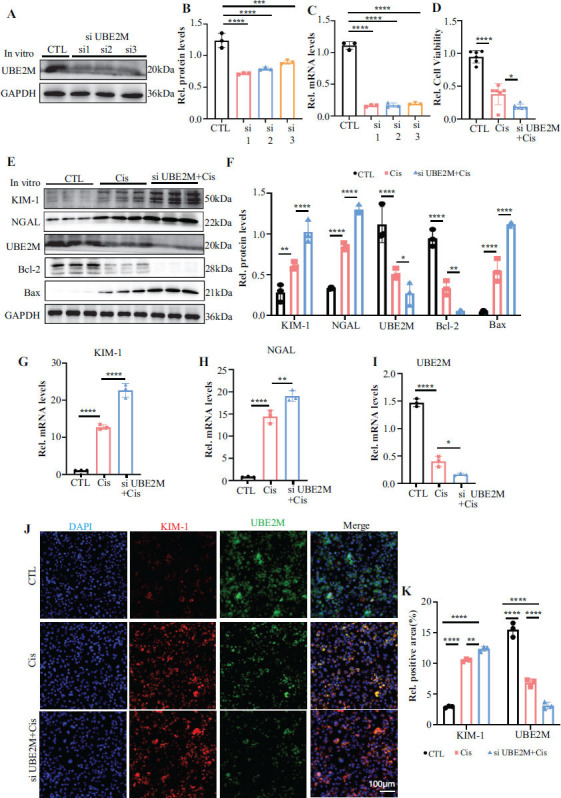
Knockdown of UBE2M aggravates cisplatin-induced injury in HK-2 cells. (**A**) Western blot analysis showing the efficiency of three different siRNAs targeting UBE2M (si1, si2, si3) compared to control (CTL) in HK-2 cells. GAPDH served as a loading control. (**B-C**) Quantification of UBE2M protein (**B**) and mRNA (**C**) levels following siRNA transfection (n = 3). (**D**) Cell viability assay demonstrating reduced survival in siUBE2M-transfected cells upon cisplatin treatment (n = 3). (**E**) Western blot analysis of KIM-1, NGAL, UBE2M, Bcl-2, and Bax protein levels in CTL, cisplatin-treated (Cis), and siUBE2M plus cisplatin-treated (siUBE2M+Cis) HK-2 cells. (**F**) Quantification of protein levels normalized to GAPDH (n = 3). (G-I) Quantification of mRNA expression levels of KIM-1 (**G**), NGAL (**H**), and UBE2M (**I**) under the indicated conditions (n = 3). (**J**) Representative immunofluorescence images of KIM-1 (red) and UBE2M (green) staining in HK-2 cells, with DAPI counterstaining (blue). Scale bar: 100 μm. (**K**) Quantification of the positive staining area for UBE2M and KIM-1 (n = 3). Data are presented as mean ± SD. *P < 0.05, **P < 0.01, ***P < 0.001, ****P < 0.0001.

To confirm the functional significance of UBE2M, we next silenced UBE2M expression and assessed its impact on cisplatin-induced injury. UBE2M knockdown by siRNA (siUBE2M) significantly reduced both UBE2M protein and transcript levels (Fig. **4A**-**C**) and aggravated cisplatin-induced cytotoxicity, as evidenced by decreased cell viability (Fig. **4D**). Compared to cisplatin alone, siUBE2M further upregulated KIM-1 and NGAL, increased Bax, and suppressed Bcl-2 expression (Fig. **4E** and **F**). qPCR data and immunostaining consistently confirmed these findings (Fig. **4G**-**K**), with the siUBE2M + Cis group showing enhanced injury marker expression and diminished UBE2M staining.

Collectively, these results demonstrate that UBE2M exerts a protective effect against cisplatin-induced tubular injury, and its loss sensitizes renal epithelial cells to damage.

### 3.5 UBE2M Regulates p53 in Cisplatin-Induced Renal Tubular Epithelial Cell Injury

Given that UBE2M overexpression alleviates, while its knockdown exacerbates, cisplatin-induced renal tubular injury (Figs. **3** and **4**), we next sought to explore the underlying molecular mechanism. p53 is a key regulator of cell apoptosis and has been implicated in cisplatin nephrotoxicity. Therefore, we next investigated whether the p53 pathway mediates the functional role of UBE2M in cisplatin-induced injury.

**Fig. (5):**
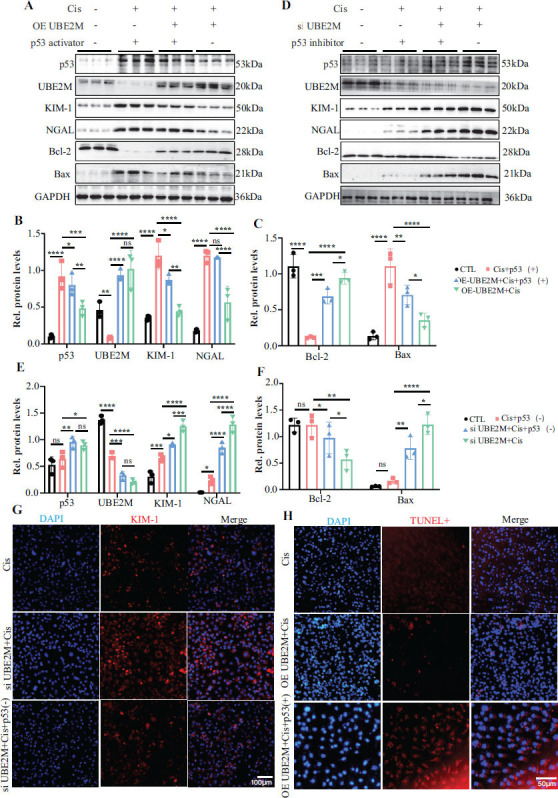
UBE2M regulates cisplatin-induced tubular injury and apoptosis *via* the p53 pathway. (**A**) Western blot analysis of p53, UBE2M, KIM-1, NGAL, Bcl-2, and Bax in HK-2 cells treated with cisplatin (Cis), UBE2M overexpression (OE-UBE2M) plus cisplatin, or OE-UBE2M combined with a p53 activator (NSC-207895, 0.5μM). GAPDH served as the loading control. (**B-C**) Quantification of relative protein levels of p53, UBE2M, KIM-1, NGAL (**B**), and Bcl-2, Bax (**C**) corresponding to the experimental groups in (A) (n = 3). (**D**) Western blot analysis of the same proteins in HK-2 cells treated with Cis, UBE2M knockdown (siUBE2M) plus cisplatin, or siUBE2M combined with a p53 inhibitor (Pifithrin-α hydrobromide, 20 μM). (**E-F**) Quantification of relative protein levels of p53, UBE2M, KIM-1, NGAL (**E**), and Bcl-2, Bax (**F**) corresponding to the experimental groups in (D) (n = 3). (**G**) Representative immunofluorescence images of KIM-1 (red) staining and nuclear counterstaining with DAPI (blue) under the indicated treatments. Scale bar: 100 μm. (**H**) TUNEL staining showing apoptotic cells (red) with DAPI (blue) nuclear counterstaining. Scale bar: 50 μm. Data are expressed as mean ± SD. *P < 0.05, **P < 0.01, ***P < 0.001, ****P < 0.0001.

In UBE2M-overexpressing cells, cisplatin treatment increased p53 levels and upregulated injury markers KIM-1 and NGAL, while reducing Bcl-2 and increasing Bax expression (Fig. **5A**). However, co-treatment with a p53 activator (NSC-207895, 0.5μM) largely abolished the protective effects of UBE2M overexpression, as shown by elevated KIM-1 and NGAL levels and a reversal of Bcl-2/Bax regulation. Quantification of protein levels confirmed this pattern (Figs. **5B** and **C**), suggesting that p53 activation negates UBE2M-mediated cytoprotection.

Conversely, in cells transfected with si UBE2M, inhibition of p53 signaling (Pifithrin-α hydrobromide, 20 μM) partially rescued the injury phenotype. As shown in Fig. (**5D**), treatment with a p53 inhibitor reduced KIM-1 and NGAL expression, increased Bcl-2 levels, and suppressed Bax expression despite UBE2M knockdown. Quantified protein levels further supported these observations (Figs. **5E** and **F**), indicating that p53 inhibition attenuates the aggravated injury caused by UBE2M deficiency.

Immunofluorescence staining revealed similar trends at the cellular level. KIM-1 expression was markedly increased in the siUBE2M + Cis group compared to the Cis group, and significantly reduced upon the addition of the p53 inhibitor (Fig. **5G**). TUNEL staining further demonstrated that UBE2M overexpression decreased apoptosis, while p53 activation reversed this effect (Fig. **5H**). These results collectively suggest that UBE2M confers protection against cisplatin-induced tubular injury at least in part by negatively regulating the p53 pathway.

## 4 DISCUSSION

In this study, we employed a genome-wide CRISPR-Cas9 screening approach to identify molecular targets involved in cisplatin-induced renal tubular epithelial cell injury. Compared to conventional hypothesis-driven approaches, CRISPR-based functional genomics allows systematic interrogation of gene function under disease-relevant stress conditions, thereby identifying previously unrecognized cellular injury regulators [18-20]. Through this high-throughput platform, we were able to uncover UBE2M, a ubiquitin-conjugating enzyme, as a novel and critical regulator of tubular damage. This methodological strength not only enhances the robustness of our findings but also provides a valuable resource for further mechanistic exploration and therapeutic development in the field of kidney injury and stress-related disorders.

UBE2M is a well-known E2 ligase involved in the ubiquitination process, which regulates protein degradation and influences various cellular processes, including inflammation, immune regulation, and apoptosis [21, 22]. Prior studies have highlighted UBE2M’s involvement in several diseases, which underscores its broader impact on cellular stress responses. In obesity models, UBE2M promotes macrophage-mediated inflammation, and its deficiency alleviates obesity-induced insulin resistance and hepatic steatosis by reducing IL-1β production [23]. In the immune system, UBE2M is vital for the function of regulatory T cells (Tregs), maintaining their homeostasis and suppressive function [24]. In cancer, particularly estrogen receptor-positive breast cancer, UBE2M has been shown to enhance tumor cell proliferation and survival [25, 26]. These diverse roles highlight UBE2M as a key integrator of stress signaling pathways, suggesting that it may similarly influence the inflammatory and apoptotic responses that drive Cis-AKI progression. Our functional studies revealed that UBE2M overexpression alleviated, whereas its knockdown aggravated, cisplatin-induced renal tubular injury.

Mechanistically, we identified the p53 signaling pathway as a critical downstream effector. The p53 pathway plays a crucial role in cisplatin-induced kidney injury. Studies have shown that p53 activation is closely associated with Cis-AKI. Specifically, p53 regulates the expression of multiple downstream genes, promoting apoptosis and inflammatory responses, thereby exacerbating kidney damage [11]. Additionally, p53 inhibits mTOR activation by upregulating specific microRNA, such as miR-199a-3p, thus influencing the progression of cisplatin-induced AKI [27]. p53 not only directly regulates apoptosis but also influences the survival and death of renal cells through interactions with other signaling pathways in Cis-AKI. For example, p53 can affect cell apoptosis and autophagy by modulating the expression of SIRT1. The activation of SIRT1 can reduce cell apoptosis by deacetylating p53, thereby counteracting cisplatin-induced kidney damage [28]. In our study, modulation of p53 activity by UBE2M suggests a novel regulatory axis that could be targeted to mitigate cisplatin-induced nephrotoxicity. Pharmacological activation of p53 negated the cytoprotective effects of UBE2M, while p53 inhibition partially rescued the aggravated injury caused by UBE2M silencing. These findings highlight the critical role of the UBE2M-p53 signaling axis in renal tubular cell survival under metabolic and genotoxic stress.

Recent studies have provided important mechanistic insights into how UBE2M regulates p53 activity. UBE2M facilitates the activation of Cullin-RING ligases (CRLs), which are known to regulate the stability of multiple substrates, including p53. Viral oncoproteins, such as E4orf6, have been shown to hijack the UBE2M-mediated neddylation machinery to activate CRL5 and promote p53 degradation, highlighting the central role of UBE2M in controlling p53 turnover *via* neddylation-dependent E3 ligase complexes [29]. Moreover, in acute myeloid leukemia models, inhibition of UBE2M or pharmacological blockade of neddylation with MLN4924 suppresses CRL activity, stabilizes p53, and enhances p53-dependent apoptosis, suggesting that UBE2M-mediated neddylation exerts a suppressive effect on p53 signaling [30]. In hepatocellular carcinoma, UBE2M has been reported to bind to MDM2, a major E3 ubiquitin ligase targeting p53, and ribosomal protein L11, an upstream regulator that stabilizes p53 under ribosomal stress. Loss of UBE2M disrupts these interactions, leading to increased p53 activation and apoptotic responses [31].

Additionally, UBE2M may also directly affect p53 post-translational modifications, such as phosphorylation or acetylation, thereby influencing its transcriptional activity. These mechanisms may collectively contribute to the modulation of p53 stability and activity during cisplatin-induced acute kidney injury (AKI). Future studies are warranted to dissect whether UBE2M directly impacts p53 turnover through these pathways in renal cells under cisplatin stress, and whether targeting UBE2M could offer a novel strategy to enhance p53-mediated protective responses in Cis-AKI.

Despite the strengths of our study, several limitations should be acknowledged. First, although UBE2M was validated as a key regulator, other candidate genes identified in the CRISPR screen were not extensively investigated and may also contribute to cisplatin nephrotoxicity. Second, our mechanistic studies were primarily performed *in vitro. In vivo* validation in animal models and human tissue samples will be crucial to establish the translational relevance of targeting UBE2M. Third, while our results implicate p53 signaling as a downstream mediator, the exact molecular interactions between UBE2M and the p53 regulatory network warrant further exploration.

## CONCLUSION 

The objective of this study was to identify novel molecular regulators of Cis-AKI and to investigate the role of UBE2M in this process. Male C57BL/6J mice were used for *in vivo* experiments, and human renal tubular epithelial HK-2 cells were used for *in vitro* studies. Key methods employed included CRISPR screening, gene overexpression and knockdown, histopathology, immunohistochemistry, TUNEL staining, ROS detection, and Western blot analysis. We identified UBE2M as a novel regulator of Cis-AKI, acting *via* modulation of the p53 pathway. These findings not only advance our understanding of the molecular mechanisms of Cis-AKI but also suggest that the UBE2M-p53 axis could represent a promising therapeutic target for preventing or treating acute kidney injury related to chemotherapy-induced stress, as well as potentially other metabolic or immune-mediated renal disorders.

## Figures and Tables

**Table 1:** Sequences of siRNA for UBE2M upregulation and downregulation.

Type	Sequence (5'-3')	Purpose
siRNA-1	Sense: AAGGUGAAGUGUGAGACAATT	UBE2M knockdown
	Antisense: UUGUCUCACACUUCACCUUTT	
siRNA-2	Sense: ACAUUGACCUCGAGGGCAATT	UBE2M knockdown
	Antisense: UUGCCCUCGAGGUCAAUGUTT	
siRNA-3	Sense: GAUGAGGGCUUCUACAAGATT	UBE2M knockdown
	Antisense: UCUUGUAGAAGCCCUCAUCTT	
CTL	Sense: UUCUCCGAACGUGUCACGUTT	Control
	Antisense: ACGUGACACGUUCGGAGAATT	

**Table 2:** List of primer sequences used for PCR in the study.

Gene Target	Forward Primer (5'-3')
Human-GAPDH-F	CTGGGCTACACTGAGCACC
Human-GAPDH-R	AAGTGGTCGTTGAGGGCAATG
Human-UBE2M-F	GGAAGCCAGTCCTTACGATAAAC
Human-UBE2M-R	CGTTCTGCTCAAACAGCCG
Human-KIM-1-F	TCCTTGGTGGGAGATAGAGG
Human-KIM-1-R	GCATTATGGGATCAGCGTTC
Human-NGAL-F	GACAACCAATTCCAGGGGAAG
Human-NGAL-R	GCATACATCTTTTGCGGGTCT
Human-p53-F	GAGGTTGGCTCTGACTGTACC
Human-p53-R	TCCGTCCCAGTAGATTACCAC
Mouse-GAPDH-F	AGGTCGGTGTGAACGGATTTG
Mouse-GAPDH-R	TGTAGACCATGTAGTTGAGGTCA
Mouse-UBE2M-F	AACCTGCCCAAGACGTGTG
Mouse-UBE2M-R	AGCTGAATACAAACTTGCCACT
Mouse-KIM-1-F	GTTAAACCAGAGATTCCCACACG
Mouse-KIM-1-R	TCTCATGGGGACAAAATGTAGTG
Mouse-NGAL-F	GGGAAATATGCACAGGTATCCTC
Mouse-NGAL-R	CATGGCGAACTGGTTGTAGTC
Mouse-p53-F	CTCTCCCCCGCAAAAGAAAAA
Mouse-p53-R	CGGAACATCTCGAAGCGTTTA

**Table 3:** Details of antibodies for western blot.

Antibody	Dilution	Supplier	Catalog Number
UBE2M	1:1000	Baijia, China	IPB13936
KIM-1(Mouse)	1:1000	R&D Systems, China	AF1817
KIM-1(Human)	1:1000	Baijia, China	IPB6471
NGAL	1:1000	Baijia, China	IPB11296
Bcl-2	1:1000	Baijia, China	IPB0022
Bax	1:1000	Baijia, China	IPB0023
p53	1:1000	Baijia, China	IPB10016
GAPDH	1:5000	Servicebio, China	GB11002

## Data Availability

All data generated or analyzed during this study are included in this published article.

## LIST OF ABBREVIATIONS

CKD: Chronic Kidney Disease
MOI: Multiplicity Of Infection
NGS: Next-Generation Sequencing
MAGeCK: Model-based Analysis of Genome-wide CRISPR-Cas9 Knockout
MEM: Minimum Essential Medium
ECL: Enhanced Chemiluminescence
IHC: Immunohistochemistry
ANOVA: Analysis of Variance
